# The Causal Fairness Field Guide: Perspectives From Social and Formal Sciences

**DOI:** 10.3389/fdata.2022.892837

**Published:** 2022-04-29

**Authors:** Alycia N. Carey, Xintao Wu

**Affiliations:** Department of Computer Science and Computer Engineering, University of Arkansas, Fayetteville, AR, United States

**Keywords:** causal modeling, fair machine learning, philosophy, sociology, law

## Abstract

Over the past several years, multiple different methods to measure the causal fairness of machine learning models have been proposed. However, despite the growing number of publications and implementations, there is still a critical lack of literature that explains the interplay of causality-based fairness notions with the social sciences of philosophy, sociology, and law. We hope to remedy this issue by accumulating and expounding upon the thoughts and discussions of causality-based fairness notions produced by both social and formal (specifically machine learning) sciences in this field guide. In addition to giving the mathematical backgrounds of several popular causality-based fair machine learning notions, we explain their connection to and interplay with the fields of philosophy and law. Further, we explore several criticisms of the current approaches to causality-based fair machine learning from a sociological viewpoint as well as from a technical standpoint. It is our hope that this field guide will help fair machine learning practitioners better understand how their causality-based fairness notions align with important humanistic values (such as fairness) and how we can, as a field, design methods and metrics to better serve oppressed and marginalized populaces.

## 1. Introduction

Due to the increasing use of machine learning in sensitive domains such as healthcare, policing, and well-fair programs, analyzing machine learning models from the lens of fairness has come into the spotlight. The majority of research efforts in fair machine learning have been focused on statistical-based measures—those that try to provide equality between different groups based on an error metric such as true positive rate. Statistical-based methods are favored since they are relatively easy to calculate and enforce. But, since statistical-based measures rely on correlation and not causation, they can only tell if an algorithm is fair based on the metric at hand. In addition, in order to take action to remedy a found fairness disparity not only would we need an explanation for how the statistic was generated, but we would also need to know how to assign responsibility and find a path to remedy the unfairness. Causality-based fairness notions allow for the analysis of the dependence between the marginalization^1^ attribute and the final decision for any cause of unfairness, which allows us to perform the tasks not possible with statistical-based measures. This fact is changing the tides of fair machine learning research, and more and more publications feature causality-based fairness notions as their focus.

Defining, implementing, and enforcing causality-based fairness in machine learning is, above all else, a sociotechnical^2^ challenge. Without viewing causality-based machine learning fairness notions from lenses of philosophy, sociology, and law, choosing and implementing a notion stays firmly technical, and does not consider societal impacts that could arise after deployment. To solve this problem, and to help fair machine learning practitioners choose correct causality-based fairness notions in an informed, societal-aware, manner, we develop the following field guide that depicts popular causality-based machine learning fairness notions through lenses of philosophy, sociology, and the law.

We note that our work is not the first to discuss the interplay of fair machine learning with the social sciences. Many works have been published over the last few years on fair machine learning (not specifically causality-based), including a handful of survey papers and textbooks that are closely aligned with this field guide (Barocas et al., 2019; Caton and Haas, 2020; Mehrabi et al., 2022). While these survey papers present mathematical aspects of mitigating bias and achieving fairness, they often only have sparse discussion (or totally omit the discussion) of philosophical and legal groundings that are important to make a sociotechnical system rather than just a technical one. Additionally, while works exist that align philosophical (Binns, 2018; Heidari et al., 2019; Khan et al., 2021; Lee et al., 2021) and legal (Barocas and Selbst, 2016; Corbett-Davies et al., 2017; Grgic-Hlaca et al., 2018; Xiang and Raji, 2019) notions with proposed fairness metrics, they often center on statistical-based fairness measures and do not speak to the emerging trend of causality-based fairness notions. Our work resolves this issue by producing a survey that presents both the social and formal discussion of causality-based fairness metrics to allow for fair machine learning practitioners to understand not only how specific fairness metrics function, but their social science groundings as well.

The rest of the field guide is as follows. We begin Section 2 by explaining the basics of causal inference followed by the introduction of two important causal frameworks that will be used throughout the rest of the field guide. In Section 3, we first present our analysis on popular causality-based fairness notions and then state their main technical pitfalls. Section 4 describes the important philosophical perspectives that serve as a foundation for many of the proposed causal fairness metrics. Next, in Section 5, we depict popular legal ideals that have a strong connection to causal fairness. In Section 6, we give critiques from a sociological viewpoint of causality-based fair machine learning. Finally, in Section 7, we present our major conclusions.

## 2. Causal Inference

The goal of standard statistical analysis is to find associations among variables in order to estimate and update probabilities of past and future events in light of new information. Causal inference analysis, on the other hand, aims to infer probabilities under conditions that are changing due to outside interventions (Pearl, 2010). Causal inference analysis (or simply causal inference) presents a formal language that allows us to draw conclusions that a specific intervention caused the observed outcome. For example, that the rain caused the grass to be wet or that taking Claritin caused your seasonal allergies to go away.

There are many different theories for understanding causality, such as regularity theories, mechanistic theories, probabilistic approaches, counterfactual reasoning, and the manipulationist approaches that house the interventionalist theories of which Pearl's structural causal model and Rubin's potential outcome frameworks belong to. In this work, we will mainly focus on the interventionalist approaches of both Pearl and Rubin as they are the most widely used frameworks for causal inference. But, we will explain the main differences between the five theories in relation to their philosophical foundations in Section 4.

### 2.1. A Primer on Causal Inference

**Figure 1:**
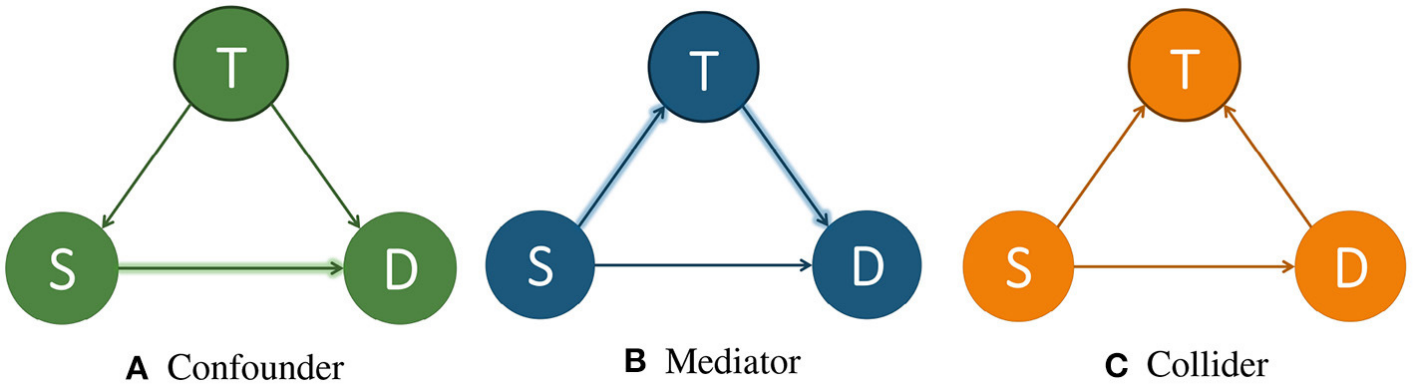
Example causal models showing *T* as a confounder, mediator, and collider. *T*, treatment; *S*, severeness; *D*, survival. In **(A)**, *T* is a confounder since it impacts both the input variable *S* and the output variable *D*. In **(B)**, *T* is a mediator since it lies between the input variable *S* and the output variable *D* in one possible path. In **(C)**, *T* is a collider since it is influenced by both *S* and *D*. An example of a direct path is shown in **(A)** by the arrow highlighted in green going from *S* to *D*. An example of an indirect path is shown in **(B)** by the arrows highlighted in blue traveling from *S* to *D* through *T*.

As the title suggests, this article focuses on causal inference based machine learning fairness notions. But, to give newcomers to the field of causal inference a solid foundation for the rest of the article, we begin by giving a short introduction of the terminology and concepts of the field. Throughout this section we will use the running example of determining whether a patient will survive a specific sickness (*D*) based on the initial severity of the disease (*S*) and the treatment administered (*T*). Three different causal diagrams showing this scenario can be seen in Figure 1. Additionally, since the discussion of causal inference here will be constrained to what is needed to understand the rest of the article, we direct interested readers to (Barocas et al., 2019; Guo et al., 2020; Yao et al., 2021) for more in-depth discussions of the topic.

There are multiple different variable types in causal inference, where each variable represents the occurrence (or non-occurrence) of an event, a property of an individual or of a population of individuals, or a quantitative value. The output variable is the particular variable that we want to affect by administering interventions, or treatments, on specific treatment variables. When administering the treatment on the treatment variable, we hold all other variable values unchanged. A variable is considered a confounder if it affects both the input and the outcome variables since it causes a spurious association^3^ between the two variables. When performing causal analysis, confounding variables must be controlled for since they can incorrectly imply that one variable caused another. An example of a confounder can be seen in Figure 1A. When a path such as *S* → *T* → *D* exists, we call *T* the mediator variable since it contributes to the overall effect of *S* on *D*. An example of this can be seen in Figure 1B. Finally, a collider is a variable that is causally influenced by two or more variables, and it is named as such since it appears that the arrow heads from the incoming variables “collide” at the node. This can be seen in Figure 1C. It is important to mention that “colliders aren't confounders” and that we should not condition on a collider since it can create a correlation between two previously uncorrelated variables (Barocas et al., 2019).

In addition to there being different types of variables, there are two main ways that one variable can cause an effect on another. The first way is a direct effect, where one variable directly affects the output variable. In order to measure the direct effect of a variable on the output variable all other possible paths (besides the direct path) need to be “disabled” or controlled. For example, in Figure 1A, we can measure the direct effect *S* has on *D* by making the treatment *T* be the same for all individuals. The other type of effect is called an indirect effect. This occurs when the effect of a variable on the output variable is transmitted through a mediator along an indirect path. An example of this can be seen in Figure 1B by the arrows highlighted in blue. In this setting, the path from *S* to *D* is mediated by the variable *T*.

Using the foundation of causal inference formed above, we can now introduce the two frameworks that are fundamental to causality-based machine learning fairness notions. The first framework is the structural causal model (SCM) framework proposed by Pearl (2009), and the second is the potential outcome (PO) framework proposed by Imbens and Rubin (2015). While we will discuss the two frameworks separately since they have different assumptions of the amount of information available, they are logically equivalent. However, we can derive a PO from a SCM, but we cannot derive a SCM from a PO alone because SCMs make more assumptions about the relationships between the variables that cannot be derived from a PO (Barocas et al., 2019).

Throughout the following discussion, and in Section 3 (which details the causality-based machine learning fairness notions), we use the following notation conventions. An uppercase letter denotes a variable, e.g., *X*; a bold uppercase letter denotes a set of variables, e.g., **X**; a lowercase letter denotes a value or a set of values of the corresponding variables, e.g., *x* and **x**; *PA*_*X*_ denotes the set of variables that directly determine the value of a variable *X* (often times called the *parents* of *X*); and pa_*X*_ denotes the values of X's parents. We also note that we will use the terms “factors” and “variables” interchangeably throughout the rest of the article.

### 2.2. Structural Causal Model

The structural causal model (SCM) was first proposed by Judea Pearl in Pearl (2009). Pearl believed that by understanding the logic behind causal thinking, we would be able to emulate it on a computer to form more realistic artificial intelligence (Pearl and Mackenzie, 2018). He proposed that causal models would give the ability to “anchor the elusive notions of science, knowledge, and data in a concrete and meaningful setting, and will enable us to see how the three work together to produce answers to difficult scientific questions,” (Pearl and Mackenzie, 2018). We recount the important details of SCMs below.

**Definition 2.1 [Structural Causal Model (Pearl, 2009)]**. *A structural causal model* M *is represented by a quadruple* 〈**U**, **V**, **F**, *P*(**U**)〉 *where*:

1. **U** *is a set of exogenous (external) variables that are determined by factors outside the model*.
2. **V** *is a set of endogenous (internal) variables that are determined by variables in* **U** ∪ **V**, *i.e.*, **V***'s values are determined by factors within the model*.
3. **F** *is a set of structural equations from* **U** ∪ **V** → **V**, i.e., *v*_*i*_ = *f*_*v*_*i*__(*pa*_*v*_*i*__, *u*_*i*_) *for each* *v*_*i*_ ∈ **V** *where* *u*_*V*_ *is a random disturbance distributed according to* *P*(*U*). *In other words*, *f*_*v*_*i*__(·) *is a structural equation that expresses the value of each endogenous variable as a function of the values of the other variables in* **U** *and* **V**.
4. *P*(**U**) *is a joint probability distribution defined over* **U**.

In general, *f*_*v*_*i*__(·) can be any type of equation. But, we will discuss *f*_*v*_*i*__(·) as a non-linear, non-parametric generalization of the standard linear equation $v_{i}=\sum_{k\in {PA}_{i}}\alpha_{ik}v_{k}+u_{i},i=1,\ldots ,n$, where α is a coefficient^4^. If all exogenous variables in **U** are assumed to be mutually independent, meaning that each variable in **U** is independent of any combination of other variables in **U**, then the causal model is called a *Markovian model*; otherwise, it is called a *semi-Markovian model*.

**Figure 2:**
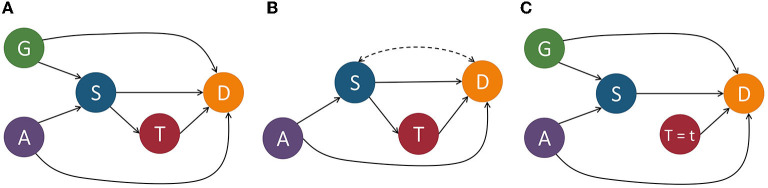
Example of a structural causal model depicting the relationships between variables that determine the survival of some disease. *G*, gender; *A*, age; *S*, severeness; *T*, treatment; *D*, survival. In **(A)**, we include the exogenous variable gender and in **(B)** we show it removed by adding a bi-directional dashed line between *S* and *D* since this is a semi-Markovian model. In **(C)**, we perform an intervention on variable *T* by setting it equal to *t*.

The causal model M is associated with a causal graph G = 〈V, E〉 where V is a set of nodes (otherwise known as vertices) and E is a set of edges. Each node of V corresponds to an endogenous variable of **V** in M. Each edge in E, denoted by a directed arrow →, points from a node *X* ∈ **U** ∪ **V** to a different node *Y* ∈ **V** if *f*_*Y*_ uses values of *X* as input. A *causal path* from *X* to *Y* is a directed path from *X* to *Y*. For example, in Figure 2A, Age(*A*) → Severeness(*S*) → Survival(*D*) is a causal path from Age to Survival. To make the causal graph easier to analyze, the exogenous variables are normally removed from the graph. In a Markovian model, exogenous variables can be directly removed without losing any vital information. In a semi-Markovian model, after removing exogenous variables, we also need to add dashed bi-directional edges between the children of correlated exogenous variables to indicate the existence of an unobserved common cause, i.e., a hidden confounder. For instance, if in Figure 2A, we treated gender as an exogenous variable, we could remove it from the graph by adding a bi-directional dashed line, as shown in Figure 2B.

Quantitatively measuring causal effects in a causal model is made possible by using the *do*-operator (Pearl, 2009) which forces some variable *X* to take on a certain value *x*. The *do*-operator can be formally denoted by *do*(*X* = *x*) or *do*(*x*). By substituting a value for another using the *do*-operator, we break the natural course of action that our model captures (Barocas et al., 2019). In a causal model M, the intervention *do*(*x*) is defined as the substituting of the structural equation *X* = *f*_*X*_(*PaX, U*_*X*_) with *X* = *x*. This change corresponds to a modified causal graph that has removed all edges coming into *X* and in turn sets *X* to *x*. An example of this can be seen in Figure 2C. For an observed variable *Y* which is affected by the intervention, its interventional variant is denoted by *Y*_*x*_. The distribution of *Y*_*x*_, also referred to as the post-intervention distribution of *Y* under *do*(*x*), is denoted by *P*(*Y*_*x*_ = *y*) or simply *P*(*y*_*x*_).

Similarly, the intervention that sets the value of a set of variables **X** to **x** is denoted by *do*(**X** = **x**). The post-intervention distribution of all other attributes **Y** = **V**\**X**, i.e., *P*(**Y** = **y**∣*do*(**X** = **x**)), or simply *P*(**y**∣*do*(**x**)), can be computed by the truncated factorization formula (Pearl, 2009):


(1) $$\begin{array}{l} P\left(\text{y}|do\left(\text{x}\right)\right)=\underset{Y\in \text{Y}}{\prod }P\left(y|PA\left(Y\right)\right)\delta_{\text{X}=\text{x}}, \end{array}$$

where δ_**X** = **x**_ assigns attributes in **X** involved in the term with the corresponding values in **x**. Specifically, the post-intervention distribution of a single attribute *Y* given an intervention on a single attribute *X* is given by:


(2) $$\begin{array}{l} P\left(y|do\left(x\right)\right)=\underset{\text{V}\backslash \left\{X,Y\right\},Y=y}{\sum }\underset{V\in \text{V}\backslash \left\{X\right\}}{\prod }P\left(v|PA\left(V\right)\right)\delta_{X=x}, \end{array}$$

where the summation is a marginalization^5^ that traverses all value combinations of **V**\{*X, Y*}. Note that *P*(*y*∣*do*(*x*)) and *P*(*y*∣*x*) are not equal. In other words, the probability distribution representing the statistical association (*P*(*y*∣*x*)) is not equivalent to the interventional distribution (*P*(*y*∣*do*(*x*))). We refer interested readers to Guo et al. (2020) for a discussion of this difference in relation to confounding bias, back-door criterion, and causal identification.

Above we mentioned that there were only two types of effects: direct and indirect. This is a slight relaxation of what can be measured in a SCM. By using the *do*-operator, we can measure multiple types of effects that one variable has on another, including: total causal effect, controlled direct effect, natural direct/indirect effect, path-specific effect, effect of treatment on the treated, counterfactual effect, and path-specific counterfactual effect. We detail their definitions below.

**Definition 2.2 [Total Causal Effect (Pearl, 2009)]**. *The total causal effect (TCE) of the value change of* *X* *from* *x*_0_ *to* *x*_1_ *on* *Y* = *y* *is given by*:


(3) $$\begin{array}{l} TCE\left(x_{1},x_{0}\right)=P\left(y_{x_{1}}\right)-P\left(y_{x_{0}}\right). \end{array}$$

The total causal effect is defined as the effect of *X* on *Y* where the intervention is transferred along all causal paths from *X* to *Y*. In contrast with the TCE, the controlled direct effect (CDE) measures the effect of *X* on *Y* while holding all the other variables fixed.

**Definition 2.3 [Controlled Direct Effect]**. *The controlled direct effect (CDE) of the value change of* *X* *from* *x*_0_ *to* *x*_1_ *on* *Y* = *y* *is given by*:


(4) $$\begin{array}{l} CDE\left(x_{1},x_{0}\right)=P\left(y_{x_{1},\text{z}}\right)-P\left(y_{x_{0},\text{z}}\right) \end{array}$$

*where* **Z** *is the set of all other variables*.

In Pearl (2013), Pearl introduced the causal mediation formula which allowed the decomposition of total causal effect into natural direct effect (NDE) and natural indirect effect (NIE).

**Definition 2.4 [Natural Direct Effect]**. *The natural direct effect (NDE) of the value change of* *X* *from* *x*_0_ *to* *x*_1_ *on* *Y* = *y* *is given by*:


(5) $$\begin{array}{l} NDE\left(x_{1},x_{0}\right)=P\left(y_{x_{1},{\text{Z}}_{x_{0}}}\right)-P\left(y_{x_{0}}\right) \end{array}$$

*where* **Z** *is the set of mediator variables and* *P*(*y*_*x*_1_, **Z**_*x*_0___) *is the probability of* *Y* = *y* *had* *X* *been* *x*_1_ *and had* **Z** *been the value it would naturally take if* *X* = *x*_0_. *In the causal graph*, *X* *is set to* *x*_1_ *in the direct path* *X* → *Y* *and is set to* *x*_0_ *in all other indirect paths*.

**Definition 2.5 [Natural Indirect Effect]**. *The natural indirect effect (NIE) of the value change of* *X* *from* *x*_0_ *to* *x*_1_ *on* *Y* = *y* *is given by*:


(6) $$\begin{array}{l} NIE\left(x_{1},x_{0}\right)=P\left(y_{x_{0},{\text{Z}}_{x_{1}}}\right)-P\left(y_{x_{0}}\right). \end{array}$$

NDE measures the direct effect of *X* on *Y* while NIE measures the indirect effect of *X* on *Y*. NDE differs from CDE since the mediators **Z** are set to **Z**_*x*_0__ in NDE and not in CDE. In other words, the mediators are set to the value that they would have naturally attained under the reference condition *X* = *x*_0_.

One main problem with NIE is that it does not enable the separation of “fair” (explainable discrimination) and “unfair” (indirect discrimination) effects (we will expound on the definitions of discrimination in the following sections). Path-specific effect (Pearl, 2009), which is an extension of TCE in the sense that the effect of the intervention is transmitted only along a subset of the causal paths from *X* to *Y*, fixes this issue. Let π denote a subset of the possible causal paths. The π-specific effect considers a counterfactual situation where the effect of *X* on *Y* with the intervention is transmitted along π, while the effect of *X* on *Y* without the intervention is transmitted along paths not in π.

**Definition 2.6 [Path-specific Effect (Avin et al., 2005)]**. *Given a causal path set π, the π-specific effect (PE_π_) of the value change of* *X* *from* *x*_0_ *to* *x*_1_ *on* *Y* = *y* *through π (with reference* *x*_0_*) is given by*:


(7) $$\begin{array}{l} {PE}_{\pi }\left(x_{1},x_{0}\right)=P\left(y_{x_{1}|\pi ,x_{0}|\bar{\pi }}\right)-P\left(y_{x_{0}}\right), \end{array}$$

*where* $P\left(Y_{x_{1}|\pi ,x_{0}|\bar{\pi }}\right)$ *represents the post-intervention distribution of* *Y* *where the effect of intervention* *do*(*x*_1_) *is transmitted only along π while the effect of reference intervention* *do*(*x*_0_) *is transmitted along the other paths*.

In addition to PE_π_ being an extension of TCE, they are further connected in that : 1) if π contains all causal paths from *X* to *Y*, then PE_π_(*x*_1_, *x*_0_) = TCE(*x*_1_, *x*_0_), and 2) for any π, we have ${\text{PE}}_{\pi }\left(x_{1},x_{0}\right)+\left(-{\text{PE}}_{\bar{\pi }}\left(x_{0},x_{1}\right)\right)=\text{TCE}\left(x_{1},x_{0}\right)$ where $\bar{\pi }$ represents the paths not in π.

Definitions 2.2 and 2.6 for TCE and PE_π_ consider the average causal effect over the *entire* population without using any prior observations. In contrast, the effect of treatment on the treated considers the effect on a sub-population of the treated group.

**Definition 2.7 [Effect of Treatment on the Treated]**. *The effect of treatment on the treated (ETT) of intervention* *X* = *x*_1_ *on* *Y* = *y* *(with baseline* *x*_0_*) is given by*:


(8) $$\begin{array}{l} {ETT}_{x_{1},x_{0}}=P\left(y_{x_{1}|x_{0}}\right)-P\left(y|x_{0}\right), \end{array}$$

*where* *P*(*y*_*x*_1_∣*x*_0__) *represents the counterfactual quantity that read as “the probability of* *Y* *would be* *y* *had* *X* *been* *x*_1_, *given that in the actual world*, *X* = *x*_0_.”

If we have certain observations about a subset of attributes **O** = **o** and use them as conditions when inferring the causal effect, then the causal inference problem becomes a *counterfactual inference* problem. This means that the causal inference is performed on the sub-population specified by **O** = **o** only. Symbolically, conditioning the distribution of *Y*_*x*_ on factual observation **O** = **o** is denoted by *P*(*y*_*x*_∣**o**). The counterfactual effect is defined as follows.

**Definition 2.8 [Counterfactual Effect (Shpitser and Pearl, 2008)]**. *Given a factual condition* **O** = **o**, *the counterfactual effect (CE) of the value change of* *X* *from* *x*_0_ *to* *x*_1_ *on* *Y* = *y* *is given by*:


(9) $$\begin{array}{l} CE\left(x_{1},x_{0}|\text{o}\right)=P\left(y_{x_{1}}|\text{o}\right)-P\left(y_{x_{0}}|\text{o}\right). \end{array}$$

In Wu et al. (2019), the authors present a general representation of causal effects, called path-specific counterfactual effect, which considers an intervention on *X* transmitted along a subset of causal paths π to *Y*, conditioning on observation **O** = **o**.

**Definition 2.9 [Path-specific Counterfactual Effect]**. *Given a factual condition* **O** = **o** *and a causal path set π, the path-specific counterfactual effect (PCE) of the value change of* *X* *from* *x*_0_ *to* *x*_1_ *on* *Y* = *y* *through π (with reference* *x*_0_*) is given by*:


(10) $$\begin{array}{l} {PCE}_{\pi }\left(x_{1},x_{0}|\text{o}\right)=P\left(y_{x_{1}|\pi ,x_{0}|\bar{\pi }}|\text{o}\right)-P\left(y_{x_{0}}|\text{o}\right). \end{array}$$

We not that in Malinsky et al. (2019), the conditional path-specific effect is written slightly different from Definition 2.9 in that, for the former, the condition is on the post-intervention distribution, and for the latter, the condition is on the pre-intervention distribution.

### 2.3. Potential Outcome Framework

The potential outcome framework (Imbens and Rubin, 2015), also known as Neyman-Rubin potential outcomes or the Rubin causal model, has been widely used in many research areas to perform causal inference since it is often easier to apply than SCM. This is because SCMs, in general, encode more assumptions about the relationships between variables and formulating a valid SCM can require domain knowledge that is not available (Barocas et al., 2019). The PO model, in contrast, is generally easier to apply since there is a broad set of statistical estimators of causal effects that can be readily applied to pure observational data.

PO refers to the outcomes one would see under each possible treatment option for a variable. Let *Y* be the outcome variable, *T* be the binary or multiple valued treatment variable, and **X** be the pre-treatment variables (covariates). Note that pre-treatment variables are the ones that are not affected by the treatment. On the other hand, the post-treatment variables, such as the intermediate outcome, are affected by the treatment.

**Definition 2.10 [Potential Outcome]**. *Given the treatment* *T* = *t* *and outcome* *Y* = *y*, *the potential outcome of the individual* *i*, *Y*_*i*_(*t*), *represents the outcome that would have been observed if the individual* *i* *had received treatment* *t*.

The potential outcome framework relies on three main assumptions:

1. Stable Unit Treatment Value Assumption (SUTVA): requires the potential outcome observation on one unit be unaffected by the particular assignment of treatments to other units.
2. Consistency Assumption: requires that the value of the potential outcomes would not change no matter how the treatment is observed or assigned through an intervention.
3. Strong Ignorability (unconfoundedness) Assumption: is equal to the assumption that there are no unobserved confounders.

Under these assumptions, causal inference methods can be applied to estimate the potential outcome and treatment effect given the information of the treatment variable and the pre-treatment variables. We refer interested readers to the survey (Yao et al., 2021) for various causal inference methods, including re-weighting, stratification, matching based, and representation based methods. In practice, only one potential outcome can be observed for each individual, while in theory, all of the different possible outcomes still exist. The observed outcome is called the factual outcome and the remaining unobserved potential outcomes are the counterfactual outcomes. The potential outcome framework aims to estimate potential outcomes under different treatment options and then calculate the treatment effect. The treatment effect can be measured at the population, treated group, subgroup, and individual levels.

As we did above for SCM, we will now recount popular ways to measure the treatment effect in PO. In addition, without loss of generality, in the following discussion we assume that the treatment variable is binary.

**Definition 2.11 [Average Treatment Effect]**. *Given the treatment* *T* = *t* *and outcome* *Y* = *y*, *the average treatment effect (ATE) is defined as*:


(11) $$\begin{array}{l} ATE=𝔼\left[Y\left(t^{\prime }\right)-Y\left(t\right)\right] \end{array}$$

*where* *Y*(*t*′) *and* *Y*(*t*) *are the potential outcome and the observed control outcome of the whole population, respectively*.

**Definition 2.12 [Average Treatment Effect on the Treated]**. *Given the treatment* *T* = *t* *and outcome* *Y* = *y*, *the average treatment effect on the treated group (ATT) is defined as*:


(12) $$\begin{array}{l} ATT=𝔼\left[Y\left(t^{\prime }\right)-Y\left(t\right)|T=t\right]. \end{array}$$

The ATE answers the question of how, on average, the outcome of interest *Y* would change if everyone in the population of interest had been assigned to a particular treatment *t*′ relative to if they had received another treatment *t*. The ATT, on the other hand, details how the average outcome would change if everyone who received one particular treatment *t* had instead received another treatment *t*′.

**Definition 2.13 [Conditional Average Treatment Effect]**. *Given the treatment* *T* = *t* *and outcome* *Y* = *y*, *the conditional average treatment effect (CATE) is defined as*:


(13) $$\begin{array}{l} CATE=𝔼\left[Y\left(t^{\prime }\right)-Y\left(t\right)|\text{W}=w\right] \end{array}$$

*where* **W** *is a subset of variables defining the subgroup*.

**Definition 2.14 [Individual Treatment Effect]**. *Given the treatment* *T* = *t* *and outcome* *Y* = *y*, *the individual treatment effect (ITE) is defined as*:


(14) $$\begin{array}{l} ITE=𝔼\left[Y_{i}\left(t^{\prime }\right)-Y_{i}\left(t\right)\right] \end{array}$$

*where* $Y_{i}\left(t^{\prime }\right)$ *and* *Y*_*i*_(*t*) *are the potential outcome and the observed control outcome of individual* *i*, *respectively*.

## 3. Causality-Based Fairness Notions

Most recent fairness notions are causality-based and reflect the now widely accepted idea that using causality is necessary to appropriately address the problem of fairness. Causality-based fairness notions differ from the statistical ones in that they are not totally based on data^6^, but consider additional knowledge about the structure of the world, in the form of a causal model. Causality-based fairness notions are developed mainly under two causal frameworks: the structural causal model (SCMs) and the potential outcome. SCMs assume that we know the complete causal graph, and hence, we are able to study the causal effect of any variable along many different paths. The potential outcome framework does not assume the availability of the causal graph and instead focuses on estimating the causal effects of treatment variables. In Table 1, we present the causal framework to which each causality-based fairness notion discussed in this section belongs. In this section, we begin by giving a short insight and overview of causality-based fairness notions, followed by a brief intermission to introduce two important statistical-fairness definitions, and then we spend the remainder of the section introducing the casual-based fairness notions, minus the last section where we state the main technical pitfalls experienced by these types of metrics.

**Table 1:** Classification of causality-based fairness notions.

**Notion**	**Association**	**SCM**	**PO**	**Intervention**	**Counterfactual**	**Y and Ŷ**
Total variation	✓					
Total causal fairness		✓		✓		
Natural direct effect		✓		✓		
Natural indirect effect		✓		✓		
Path-specific causal fairness		✓		✓		
Direct causal fairness		✓		✓		
Indirect causal fairness		✓		✓		
Counterfactual fairness		✓			✓	
Counterfactual direct effect		✓			✓	
Counterfactual indirect effect		✓			✓	
Path-specific counterfactual fairness		✓			✓	
Proxy fairness		✓		✓		
Justifiable fairness		✓		✓		
Counterfactual direct error rate		✓			✓	✓
Counterfactual indirect error rate		✓			✓	✓
Individual equalized counterfactual odds		✓			✓	✓
Fair on average causal effect			✓	✓		
Fair on average causal effect on the treated			✓		✓	
Equal effort fairness			✓		✓	

*SCM, structure causal model; PO, potential outcome. The last column describes whether the fairness notion involves both Y and Ŷ in their counterfactual quantity. A checkmark means that the causality-based fairness notion falls within the given category. For example, total causal fairness belongs to both the SCM framework and the intervention rung of Pearl's ladder of causation*.

**Figure 3:**
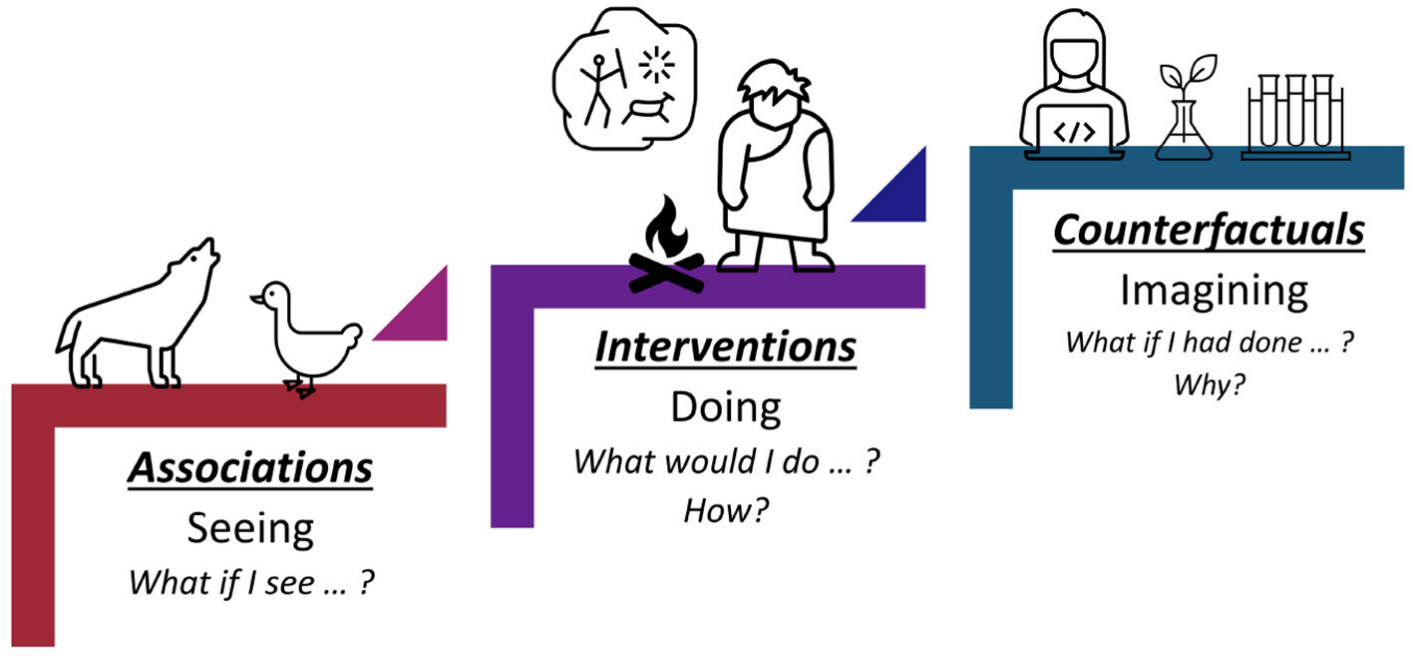
Pearl's Ladder of Causation. The first rung, associations, only allows predictions based on passive observations. The second rung, interventions, not only relies on seeing, but also changing what is. Rung three, counterfactuals, deals with the imaginary, or what might have been.

In Pearl (2019), Pearl presented the causal hierarchy through the Ladder of Causation, as shown in Figure 3. The Ladder of Causation has the 3 rungs: association, intervention, and counterfactual. The first rung, associations, can be inferred directly from the observed data using conditional probabilities and conditional expectations (i.e., a probabilistic theory, see Section 4). The intervention rung involves not only seeing what is, but also changing what we see. Interventional questions deal with *P*(*y*∣*do*(*x*), *z*) which stands for “the probability of *Y* = *y*, given that we intervene and set the values of *X* to *x* and subsequently observe event *Z* = *z*.” Interventional questions cannot be answered from pure observational data alone. They can be estimated experimentally from randomized trials or analytically using causal Bayesian networks. The top rung invokes counterfactuals and deals with $P\left(y_{x}|x^{\prime },y^{\prime }\right)$ which stands for “the probability that event *Y* = *y* would be observed had *X* been *x*, given that we actually observed *X* to be *x*′ and *Y* to be *y*′.” Such questions can be computed only when the model is based on functional relations or is structural. In Table 1, we also show the causal hierarchical level that each causality-based fairness notion aligns with.

**Figure 4:**
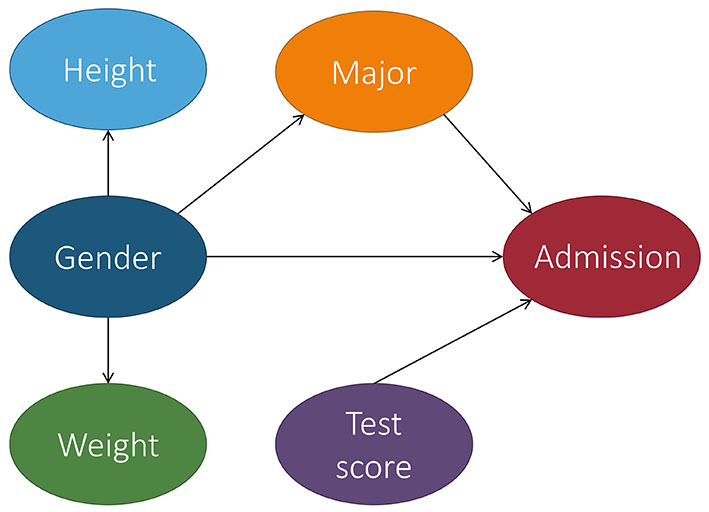
Causal graph of the college admission example used throughout this section. Let gender be the marginalization attribute and Female be the marginalized class. For simplicity, we consider gender to be binary, but we recognize that this is not the case in real life.

In the context of fair machine learning, we use *S* ∈ {*s*^+^, *s*^−^} to denote the marginalization attribute, *Y* ∈ {*y*^+^, *y*^−^} to denote the decision, and **X** to denote a set of non-marginalization attributes. The underlying mechanism of the population over the space *S* × **X** × *Y* is represented by a causal model M, which is associated with a causal graph G. Figure 4 shows a causal graph that will be used to illustrate fairness notions throughout this section. With M, we want to reason about counterfactual queries, e.g., “what would the prediction have been for this individual if their marginalization attribute value changed?” A historical dataset D is drawn from the population, which is used to construct a predictor *h*:**X**, *S* → Ŷ. Note that the input of the predictor can be a subset of **X**, *S* and we use $\hat{PA}$ to denote the set of input features of the predictor when introducing counterfactual error rate in Section 3.9. The causal model for the population over space *S* × **X** × Ŷ can be considered the same as M, except that the function *f*_*Y*_ is replaced with a predictor *h*. Most fairness notions involve either *Y* or Ŷ in their counterfactual quantity and, roughly speaking, they correspond to statistical parity (a statistical-based notion introduced below). A few fairness notions, e.g., counterfactual direct error rate (Zhang and Bareinboim, 2018a), correspond to the concept of equalized odds (also explained below) and involve both *Y* and Ŷ in their counterfactual quantity. We also mark if a notion uses *Y* and/or Ŷ in Table 1. We note that for all of the fairness notions presented here, there actually exists two versions—strict and relaxed. The strict version means there is absolutely no discrimination effect (i.e., no wiggle room), whereas the relaxed version often compares the causal effect with τ, a user-defined threshold for discrimination (i.e., wiggle room). Despite having two approaches, for simplicity, we adhere to the strict version when introducing each fairness notion in the discussion below.

### 3.1. Statistical-Based Fairness Notions

Despite the claims we have made against using statistical-based fairness notions so far, we do wish to introduce two popular metrics: statistical parity and equalized odds. Our reasoning of doing so is two-fold: (1) these two statistical notions are closely tied to several causality-based fairness notions, and (2) they present a clear picture of why causality-based machine learning fairness notions are preferred over statistical ones.

We will begin by describing statistical parity, which also goes by the names demographic parity and group fairness. As the name implies, it requires that there is an equal probability for both individuals in the marginalized and non-marginalized groups to be assigned to the positive class (Dwork et al., 2011; Kusner et al., 2017). Notationally, group fairness can be written as:


(15) $$\begin{array}{l} P\left(\hat{Y}=1|S=0\right)=P\left(\hat{Y}=1|S=1\right) \end{array}$$

where Ŷ is the predicted outcome and *S* is the marginalization variable.

Barocas, Hardt, and Narayanan note that while statistical parity aligns well with how humans reason about fairness, several draw-backs exists (Barocas et al., 2019). Namely, that it ignores any correlation between the marginalization attributes and the target variable *Y* which constrains the construction of a perfect prediction model. Additionally, it enables laziness. In other words, it allows situations where qualified people are carefully selected for one group (e.g., non-marginalized), while random people are selected for the other (marginalized). Further, it allows the trade of false negatives for false positives, meaning that neither of these rates are considered more important, which is false in many circumstances (Barocas and Hardt, 2017).

The fairness metric of equalized odds is also known as conditional procedure accuracy equality and disparate mistreatment. Whereas, statistical parity requires that the probability of being classified as positive is the same for all groups, equalized odds requires that true and false positive rates are similar across different groups (Moritz et al., 2016). In other words, equalized odds enforces equality among individuals who have similar outcomes. It can be written as:


(16) $$\begin{array}{l} P(\hat{Y}=1|Y=y\cap S=0)\\ \;=P(\hat{Y}=1|Y=y\cap S=1)\text{ for }y\in \{0,1\} \end{array}$$

where Ŷ is the predicted outcome, *Y* is the actual outcome, and *S* is the marginalization attribute.

### 3.2. Total, Natural Direct, and Natural Indirect Causal Fairness

We now move into our main discussion of the causality-based fairness notions, starting with a discussion of total, natural direct, and natural indirect causal fairness. Discrimination can be viewed as the causal effect of *S* on *Y*. Total causal fairness answers the question of if the marginalization attribute *S* changed (e.g., changing from marginalized group *s*^−^ to non-marginalized group *s*^+^), how would the outcome *Y* change on average? A straightforward strategy to answer this question is to measure the average causal effect of *S* on *Y* when *S* changes from *s*^−^ to *s*^+^, an approach called total causal fairness.

**Definition 3.1 [Total Causal Fairness]**. *Given the marginalization attribute* *S* *and decision* *Y*, *we achieve total causal fairness if*:


(17) $$\begin{array}{l} TCE\left(s_{1},s_{0}\right)=P\left(y_{s_{1}}\right)-P\left(y_{s_{0}}\right)=0 \end{array}$$

*where* $s_{1},s_{0}\in \left\{s^{+},s^{-}\right\}$.

For instance, based on Figure 4, TCE would report the average causal effect that being Female had on a student's outcome of admission.

Additionally, the causal effect of *S* on *Y* does not only include the direct discriminatory effect, but it also includes the indirect discriminatory effect and the explainable effect. In Pearl (2013), Pearl proposed the use of NDE and NIE to measure the direct and indirect discrimination. Recall from Definitions 2.4, 2.5 that NDE(*s*_1_, *s*_0_) = *P*(*y*_*s*_1_, **Z**_*s*_0___) − *P*(*y*_*s*_0__) and NIE(*s*_1_, *s*_0_) = *P*(*y*_*s*_0_, **Z**_*s*_1___) − *P*(*y*_*s*_0__) where **Z** is the set of mediator variables. When applied to the example in Figure 4, the mediator variable could be the major. *P*(*y*_*s*_1_, **Z**_*s*_0___) in NDE is the probability of *Y* = *y* had *S* been *s*_1_ and had **Z** been the value it would naturally take if *S* = *s*_0_. In other words, based on the example, *P*(*y*_*s*_1_, **Z**_*s*_0___) would be the probability of being admitted when changing the gender to be Male while keeping the major the same. Similarly, NIE measures the indirect effect of *S* on *Y*. However, NIE does not distinguish between explainable and indirect discrimination.

### 3.3. Path-Specific Causal Fairness

In Zhang et al. (2017), Zhang et al. introduced path-specific causal fairness based on the path-specific causal effect (Pearl, 2009) notion presented in Definition 2.9. Different from total, natural direct, and natural indirect causal effects, the path-specific causal effect is based on graph properties of the causal graph (where the others were based on probabilities), and characterizes the causal effect in term of specific paths.

**Definition 3.2 [Path-Specific Causal Fairness]**. *Given the marginalization attribute* *S*, *decision* *Y*, *and* *redlining attributes* **R** *(i.e., a set of attributes in* **X** *that cannot be legally justified if used in decision-making), define π_*d*_ as the path set that contains some paths from* *S* *to* *Y*. *We achieve path-specific causal fairness if*:


(18) $$\begin{array}{l} {PE}_{\pi }\left(s_{1},s_{0}\right)=P\left(y_{s_{1}|\pi ,s_{0}|\bar{\pi }}\right)-P\left(s_{x_{0}}\right)=0 \end{array}$$

*where* $s_{1},s_{0}\in \left\{s^{+},s^{-}\right\}$. *Specifically, define π*_*d*_ *as the path set that contains only* *S* → *Y* *and define π*_*i*_ *as the path set that contains all the causal paths from* *S* *to* *Y* *which pass through some redlining attributes of* **R**. *We achieve direct causal fairness if PE*_π_*d*__(*s*_1_, *s*_0_) = 0, *and indirect causal fairness if PE*_π_*i*__(*s*_1_, *s*_0_) = 0.

Direct discrimination considers the causal effect transmitted along the direct path from *S* to *Y*, i.e., *S* → *Y*. The physical meaning of *PE*_π_*d*__(*s*_1_, *s*_0_) can be explained as the expected change in decisions of individuals from marginalized group *s*_0_, if the decision makers are told that these individuals were from the non-marginalized group *s*_1_. When applied to the example in Figure 4, it means that the expected change in admission of applicants is actually from the marginalized group (e.g., Female), when the admission office is instructed to treat the applicants as from the non-marginalized group (e.g., Male).

Indirect discrimination considers the causal effect transmitted along all the indirect paths from *S* to *Y* that contain the redlining attributes. The physical meaning of *PE*_π_*i*__(*s*_1_, *s*_0_) is the expected change in decisions of individuals from marginalized group *s*_0_, if the values of the redlining attributes in the profiles of these individuals were changed as if they were from the non-marginalized group *s*_1_. When applied to the example in Figure 4, it means the expected change in admission of the marginalized group if they had the same gender makeups shown in the major as the non-marginalized group.

The following propositions (Zhang et al., 2017) further show two properties of the path-specific effect metrics.

**Proposition 3.1**. *If path set π contains all causal paths from* *S* *to* *Y* *and* *S* *has no parent in* G, *then we have*:


(19) $$\begin{array}{l} {PE}_{\pi }\left(s_{1},s_{0}\right)=TCE\left(s_{1},s_{0}\right)=P\left(y^{+}|s_{1}\right)-P\left(y^{+}|s_{0}\right). \end{array}$$

$P\left(y^{+}|s_{1}\right)-P\left(y^{+}|s_{0}\right)$ is known as the *risk difference* (a measure of statistical parity). Therefore, the path-specific effect metrics can be considered as an extension to the risk difference (and statistical parity) for explicitly distinguishing the discriminatory effects of direct and indirect discrimination from the total causal effect.

**Proposition 3.2**. *For any path sets π*_*d*_ *and* π_*i*_, *we do not necessarily have*:


(20) $$\begin{array}{l} {PE}_{\pi_{d}}\left(s_{1},s_{0}\right)+{PE}_{\pi_{i}}\left(s_{1},s_{0}\right)={PE}_{\pi_{d}\cup \pi_{i}}\left(s_{1},s_{0}\right). \end{array}$$

This implies that there might not be a linear connection between direct and indirect discrimination.

### 3.4. Counterfactual Fairness

In Sections 3.2 and 3.3, the intervention is performed on the whole population. These metrics deal with effects on an entire population, or on the average individual from a population. But, up to this point we have not talked about “personalized causation”—or causation at the level of particular events of individuals (Pearl and Mackenzie, 2018). Counterfactuals will allow us to do so. If we infer the post-intervention distribution while conditioning on certain individuals, or groups specified by a subset of observed variables, the inferred quantity will involve two worlds simultaneously: the real world represented by causal model M, as well as the counterfactual world M_*x*_. Such causal inference problems are called counterfactual inference, and the distribution of *Y*_*x*_ conditioning on the real world observation **O** = **o** is denoted by *P*(*y*_*x*_ ∣ **o**).

In Kusner et al. (2017), Kusner et al. defined counterfactual fairness to be the case where the outcome would have remained the same had the marginalization attribute of an individual or a group been different, and all other attributes been equal.

**Definition 3.3 [Counterfactual Fairness]**. *Given a factual condition* ***O*** = ***o*** *where* ***O*** ⊆ {*S*, **X**, *Y*}, *we achieve counterfactual fairness if*:


(21) $$\begin{array}{l} CE\left(s_{1},s_{0}|o\right)=P\left(y_{s_{1}}|\text{o}\right)-P\left(y_{s_{0}}|\text{o}\right)=0 \end{array}$$

*where* $s_{1},s_{0}\in \left\{s^{+},s^{-}\right\}$.

Note that we can simply define a classifier as counterfactually fair by replacing outcome *Y* with the predictor Ŷ in the above equation. The meaning of counterfactual fairness can be interpreted as follows when applied to the example in Figure 4. Applicants are applying for admission and a predictive model is used to make the decision Ŷ. We concern ourselves with an individual from marginalized group *s*_0_ who is specified by a profile **o**. The probability of the individual to get a positive decision is *P*(ŷ ∣ *s*_0_, **o**), which is equivalent to *P*(ŷ_*s*_0__ ∣ *s*_0_, **o**) since the intervention makes no change to *S*'s value of that individual. Now assume the value of *S* for the individual had been changed from *s*_0_ to *s*_1_. The probability of the individual to get a positive decision after the hypothetical change is given by *P*(ŷ_*s*_1__ ∣ *s*_0_, **o**). Therefore, if the two probabilities *P*(ŷ_*s*_0__ ∣ *s*_0_, **o**) and *P*(ŷ_*s*_1__ ∣ *s*_0_, **o**) are identical, we can claim the individual is treated fairly as if they had been from the other group.

### 3.5. Counterfactual Effects

In Zhang and Bareinboim (2018b), Zhang and Bareinboim introduced three fine-grained measures of the transmission of change from stimulus to effect called the counterfactual direct, indirect, and spurious effects. Throughout Section 3.5, we use **W** to denote all the observed intermediate variables between *S* and *Y* and use the group with *S* = *s*_0_ as the baseline to measure changes of the outcome.

**Definition 3.4 [Counterfactual Direct Effect]**. *Given a SCM, the counterfactual direct effect (Ctf-DE) of intervention* *S* = *s*_1_ *on* *Y* *(with baseline* *s*_0_*) conditioned on* *S* = *s* *is defined as*:


(22) $$\begin{array}{l} Ctf\text{-}{DE}_{s_{0},s_{1}}(y|s)=P(y_{s_{1},W_{s_{0}}}|s)-P(y_{s_{0}}|s). \end{array}$$

*Y*_*s*_1_, **W**_*s*_0___ = *y*∣*S* = *s* is a more involved counterfactual compared to NDE and can be read as “the value *Y* would be had *S* been *s*_1_, while **W** is kept at the same value that it would have attained had *S* been *s*_0_, given that *S* was actually equal to *s*.” In terms of Figure 4, *Y*_*s*_1_, **W**_*s*_0___ = *y*∣*S* = *s* means the admission decision for a Female student if they had actually been Male, while keeping all intermediate variables the same, when given that the student's gender is actually *s* (meaning Male or Female).

**Definition 3.5 [Counterfactual Indirect Effect]**. *Given a SCM, the counterfactual indirect effect (Ctf-IE) of intervention* *S* = *s*_1_ *on* *Y* *(with baseline* *s*_0_*) conditioned on* *S* = *s* *is defined as*:


(23) $$\begin{array}{l} {Ctf\text{-}IE}_{s_{0},s_{1}}\left(y|s\right)=P\left(y_{s_{0},{\text{W}}_{s_{1}}}|s\right)-P\left(y_{s_{0}}|s\right). \end{array}$$

Ctf-IE measures changes in the probability of the outcome *Y* being *y* had *S* been *s*_0_, while changing **W** to whatever level it would have naturally obtained had *S* been *s*_1_, in particular, for the individuals in which *S* = *s*_0_. In terms of Figure 4, this means the probability of admission for a Female student based on the intermediate variable values that would be obtained if they were Male (e.g., ratio of Males applying to the major).

**Definition 3.6 [Counterfactual Spurious Effect]**. *Given a SCM, the counterfactual spurious effect (Ctf-SE) of* *S* = *s*_1_ *on* *Y* = *y* *(with baseline* *s*_0_*) is defined as*:


(24) $$\begin{array}{l} {Ctf\text{-}SE}_{s_{0},s_{1}}\left(y\right)=P\left(y_{s_{0}}|s_{1}\right)-P\left(y|s_{0}\right). \end{array}$$

Ctf-SE_*s*_0_, *s*_1__(*y*) measures the difference in the outcome *Y* = *y* had *S* been *s*_0_ for the individuals that would naturally choose *S* to be *s*_0_ vs. *s*_1_. In other words, it measures the difference in the admission decision had the marginalization attribute been set to Female for the students that were actually Female vs. Male.

**Proposition 3.3**. *For a SCM, if* *S* *has no direct (indirect) causal path connecting* *Y* *in the causal graph, then Ctf-DE*_*s*_0_, *s*_1__(*y*∣*s*) = 0 *(Ctf-IE*_*s*_0_, *s*_1__(*y*∣*s*) = 0*) for any* *s*, *y*; *if* *S* *has no back-door*^7^ *path connecting* *Y* *in the causal graph, then Ctf-SE*_*s*_0_, *s*_1__(*y*) = 0 *for any* *y*.

Building on these measures, Zhang and Bareinboim derived the causal explanation formula for the disparities observed in the total variation. Recall that the total variation is simply the difference between the conditional distributions of *Y* when observing *S* changing from *s*_0_ to *s*_1_.

**Definition 3.7 [Total Variation]**. *The total variation (TV) of* *S* = *s*_1_ *on* *Y* = *y* *(with baseline* *s*_0_*) is given by*:


(25) $$\begin{array}{l} {TV}_{s_{0},s_{1}}\left(y\right)=P\left(y|s_{1}\right)-P\left(y|s_{0}\right). \end{array}$$

In regard to Figure 4, the TV would be the probability of the outcome given that the student was Male minus the probability of the outcome given that the student was Female., i.e., the difference in their overall probabilities of being admitted.

**Theorem 3.1 [Causal Explanation Formula]**. *For any* *s*_0_, *s*_1_, *y*, *the total variation, counterfactual spurious, direct, and indirect effects obey the following relationship*:


(26) $$\begin{array}{l} {TV}_{s_{0},s_{1}}\left(y\right)={Ctf\text{-}SE}_{s_{0},s_{1}}\left(y\right)+{Ctf\text{-}IE}_{s_{0},s_{1}}\left(y|s_{1}\right)-{Ctf\text{-}SD}_{s_{1},s_{0}}\left(y|s_{1}\right), \end{array}$$

(27) $$\begin{array}{l} {TV}_{s_{0},s_{1}}\left(y\right)={Ctf\text{-}DE}_{s_{0},s_{1}}\left(y|s_{0}\right)-{Ctf\text{-}SE}_{s_{1},s_{0}}\left(y\right)-{Ctf\text{-}IE}_{s_{1},s_{0}}\left(y|s_{0}\right). \end{array}$$

Theorem 3.1 allows the machine learning designer to quantitatively evaluate fairness and explain the total observed disparity of a decision through different discriminatory mechanisms. For example, the first formula shows that the total disparity experienced by the individuals who have naturally attained *s*_1_ (relative to *s*_0_, in other words, students who were naturally Male over Female) is equal to the disparity associated with spurious discrimination, plus the advantage it lost due to indirect discrimination, minus the advantage it would have gained without direct discrimination.

### 3.6. Path-Specific Counterfactual Fairness

In Wu et al. (2019), Wu et al. proposed path-specific counterfactual fairness (PC fairness) that covers the previously mentioned fairness notions. Letting Π be all causal paths from *S* to *Y* in the causal graph and π be a subset of Π, the path-specific counterfactual fairness metric is defined as follows.

**Definition 3.8 [Path-specific Counterfactual Fairness (PC Fairness)]**. *Given a factual condition* ***O*** = ***o*** *where* ***O*** ⊆ {*S*, **X**, *Y*} *and a causal path set π, we achieve the PC fairness if*:


(28) $$\begin{array}{l} {PCE}_{\pi }\left(s_{1},s_{0}|o\right)=P\left(y_{s_{1}|\pi ,s_{0}|\bar{\pi }}|\text{o}\right)-P\left(y_{s_{0}}|\text{o}\right)=0 \end{array}$$

*where* $s_{1},s_{0}\in \left\{s^{+},s^{-}\right\}$.

In order to achieve path-specific counterfactual fairness in the running example, the application decision system needs to be able to discern the causal effect of the applicants gender being Female along the fair and unfair pathways, and to disregard the effect along the pathways that are unfair.

We point out that we can simply define the PC Fairness on a classifier by replacing outcome *Y* with the predictor Ŷ in the above equation. Previous causality-based fairness notions can be expressed as special cases of the PC fairness based on the value of **O** (e.g., ∅ or *S*, **X**) and the value of π (e.g., Π or π_*d*_). Their connections are summarized in Table 2, where π_*d*_ contains the direct edge from *S* to Ŷ, and π_*i*_ is a path set that contains all causal paths passing through any redlining attributes. The notion of PC fairness also resolves new types of fairness, e.g., individual indirect fairness, which means discrimination along the indirect paths for a particular individual. Formally, individual indirect fairness can be directly defined and analyzed using PC fairness by letting **O** = {*S*, **X**} and π = π_*i*_.

**Table 2:** Connection between Path-specific Counterfactual Fairness (PC Fairness) and other fairness notions.

**Description**	**Relating to PC fairness**
Total causal fairness	**O** = ∅ and π = Π
Direct causal fairness	**O** = ∅ and π = π_*d*_ = {*S* → Ŷ}
Indirect causal fairness	**O** = ∅ and π = π_*i*_⊂Π
Counterfactual fairness	**O** = {*S*, **X**} and π = Π
Counterfactual direct effect (Ctf-DE)	**O** = {*S, Y*} and π = π_*d*_
Counterfactual indirect effect (Ctf-IE)	**O** = {*S, Y*} and π_*i*_

### 3.7. Proxy Fairness

**Figure 5:**
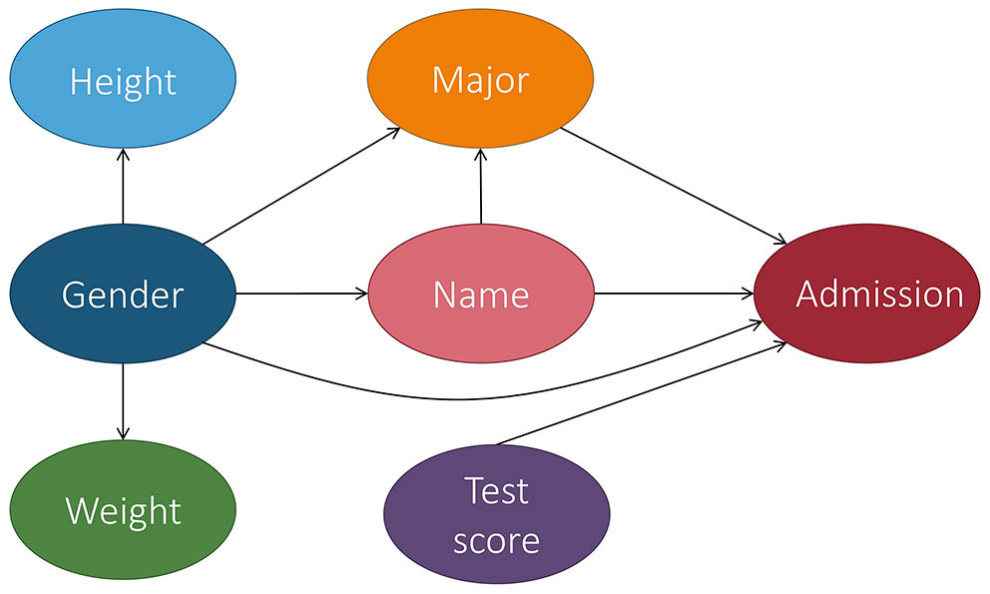
Extension of Figure 4 in which we add a proxy variable: name. Name is significantly correlated with the marginalization attribute gender since a person's name is often chosen based on their gender.

In Kilbertus et al. (2017), Kilbertus et al. proposed proxy fairness. A proxy is a descendant of *S* in the causal graph whose observable quantity is significantly correlated with *S*, but should not affect the prediction. An example of a proxy variable in our running admission case can be seen in Figure 5.

**Definition 3.9 [Proxy Discrimination]**. *A predictor Ŷ exhibits no proxy discrimination based on a proxy* *P* *if for all* *p, p*′ *we have*:


(29) $$\begin{array}{l} P\left(ŷ|do\left(P=p\right)\right)=P\left(Ŷ|do\left(P=p^{\prime }\right)\right) \end{array}$$

Intuitively, a predictor satisfies proxy fairness if the distribution of Ŷ under two interventional regimes in which *P* set to *p* and *p*′ is the same. Kilbertus et al. (2017) presented the conditions and developed procedures to remove proxy discrimination given the structural equation model.

### 3.8. Justifiable Fairness

In Salimi et al. (2019), Salimi et al. presented a pre-processing approach for removing the effect of any discriminatory causal relationship between the marginalization attribute and classifier predictions by manipulating the training data to be non-discriminatory. The repaired training data can be seen as a sample from a hypothetical fair world.

**Definition 3.10 [K-fair]**. *For a give set of variables* **K**, *a decision function is said to be* **K**-*fair with regards to* *S* *if, for any context* **K** = **k** *and any outcome* *Y* = *y*, *P*(*y*_*s*_0_, **k**_) = *P*(*y*_*s*_1_, **k**_).

Note that the notion of **K**-fair intervenes on both the marginalization attribute *S* and variables **K**. It is more fine-grained than proxy fairness, but it does not attempt to capture fairness at the individual level. The authors further introduced justifiable fairness for applications where the user can specify admissible (deconfounding) variables through which it is permissible for the marginalization attribute to influence the outcome. In our example from Figure 4, the admissible variable is the major.

**Definition 3.11 [Justifiable Fairness]**. *A fairness application is justifiable fair if it is* **K**-*fair with regarding to all supersets* **K** ⊇ **A** *where* **A** *is the set of admissible variables*.

Different from previous causality-based fairness notions, which require the presence of the underlying causal model, the justifiable fairness notion is based solely on the notion of intervention. The user only requires specification of a set of admissible variables and does not need to have a causal graph. The authors also introduced a sufficient condition for testing justifiable fairness that does not require access to the causal graph. However, with the presence of the causal graph, if all directed paths from *S* to *Y* go through an admissible attribute in **A**, then the algorithm is justifiably fair. If the probability distribution is faithful to the causal graph, the converse also holds. This means that our running example is not justifiably fair since the paths from gender to admission has two paths: gender → major → admission and gender → admission.

### 3.9. Counterfactual Error Rate

Zhang and Bareinboim (2018a) developed a causal framework to link the disparities realized through equalized odds (EO) and the causal mechanisms by which the marginalization attribute *S* affects change in the prediction Ŷ. EO, also referred to as error rate balance, considers both the ground truth outcome *Y* and predicted outcome Ŷ. EO achieves fairness through the balance of the misclassification rates (false positive and negative) across different demographic groups. They introduced a family of counterfactual measures that allows one to explain the misclassification disparities in terms of the direct, indirect, and spurious paths from *S* to Ŷ on a structural causal model. Different from all previously discussed causality-based fairness notions, counterfactual error rate considers both *Y* and Ŷ in their counterfactual quantity.

**Definition 3.12 [Counterfactual Direct Error Rate]**. *Given a SCM and a classifier* $ŷ=f\left(\hat{pa}\right)$ *where* $\hat{PA}$ *is a set of input features of the predictor, the counterfactual direct error rate (ER*^*d*^*) for a sub-population *s, y* (with prediction ŷ ≠ *y*) is defined as*:


(30) $$\begin{array}{l} {ER}_{s_{0},s_{1}}^{d}\left(ŷ|s,y\right)=P\left({ŷ}_{s_{1},y,{\left(\hat{PA}\backslash S\right)}_{s_{0},y}}|s,y\right)-P\left({ŷ}_{s_{0},y}|s,y\right). \end{array}$$

For an individual with the marginalization attribute *S* = *s* and the true outcome *Y* = *y*, the counterfactual direct error rate calculates the difference of two terms. The first term is the prediction Ŷ had *S* been *s*_1_, while keeping all the other features $\hat{PA}\backslash S$ at the level that they would attain had *S* = *s*_0_ and *Y* = *y*, whereas the second term is the prediction Ŷ the individual would receive had *S* been *s*_0_ and *Y* been *y*.

**Definition 3.13 [Counterfactual Indirect Error Rate]**. *Given a SCM and a classifier* $ŷ=f\left(\hat{pa}\right)$, *the counterfactual indirect error rate (ER*^*i*^*) for a sub-population* *s, y* *(with prediction ŷ ≠ y) is defined as*:


(31) $$\begin{array}{l} {ER}_{s_{0},s_{1}}^{i}\left(ŷ|s,y\right)=P\left({ŷ}_{s_{0},y,{\left(\hat{PA}\backslash S\right)}_{s_{1},y}}|s,y\right)-P\left({ŷ}_{s_{0},y}|s,y\right). \end{array}$$

**Definition 3.14 [Counterfactual Spurious Error Rate]**. *Given a SCM and a classifier* $ŷ=f\left(\hat{pa}\right)$, *the counterfactual spurious error rate (ER*^*s*^*) for a sub-population* *s, y* *(with prediction ŷ ≠ y) is defined as*:


(32) $$\begin{array}{l} {ER}_{s_{0},s_{1}}^{s}\left(ŷ|y\right)=P\left({ŷ}_{s_{0},y}|s_{1},y\right)-P\left({ŷ}_{s_{0},y}|s_{0},y\right). \end{array}$$

The counterfactual spurious error rate can be read as “for two demographics *s*_0_, *s*_1_ with the same true outcome *Y* = *y*, how would the prediction Ŷ differ had they both been *s*_0_, *y*?” For a graphical depiction of these measures, we refer interested reader to the tutorial by Bareinboim et al.

Building on these measures, Zhang and Bareinboim (2018a) derived the causal explanation formula for the error rate balance. The equalized odds notion constrains the classification algorithm such that its disparate error rate is equal to zero across different demographics.

**Definition 3.15 [Error Rate Balance]**. *The error rate (ER) balance is given by*:


(33) $$\begin{array}{l} {ER}_{s_{0},s_{1}}\left(ŷ|y\right)=P\left(ŷ|s_{1},y\right)-P\left(ŷ|s_{0},y\right). \end{array}$$

**Theorem 3.2 [Causal Explanation Formula of Equalized Odds]**. *For any* *s*_0_, *s*_1_, ŷ, *y*, *we have the following relationship*:


(34) $$\begin{array}{l} {ER}_{s_{0},s_{1}}\left(ŷ|y\right)={ER}_{s_{0},s_{1}}^{d}\left(ŷ|s_{0},y\right)-{ER}_{s_{1},s_{0}}^{i}\left(ŷ|s_{0},y\right)-{ER}_{s_{1},s_{0}}^{s}\left(ŷ|y\right). \end{array}$$

The above theorem shows that the total disparate error rate can be decomposed into terms, each of which estimates the adverse impact of its corresponding discriminatory mechanism.

### 3.10. Individual Equalized Counterfactual Odds

In Pfohl et al. (2019), Pfohl et al. proposed the notion of individual equalized counterfactual odds that is an extension of counterfactual fairness and equalized odds. The notion is motivated by clinical risk prediction and aims to achieve equal benefit across different demographic groups.

**Definition 3.16 [Individual Equalized Counterfactual Odds]**. *Given a factual condition* **O** = **o** *where* **O** ⊆ {**X**, *Y*}, *predictor Ŷ achieves the individual equalized counterfactual odds if*:


(35) $$\begin{array}{l} P\left({ŷ}_{s_{1}}|\text{o},y_{s_{1}},s_{0}\right)-P\left({ŷ}_{s_{0}}|\text{o},y_{s_{0}},s_{0}\right)=0 \end{array}$$

*where* $s_{1},s_{0}\in \left\{s^{+},s^{-}\right\}$.

The notion implies that the predictor must be counterfactually fair given the outcome *Y* matching the counterfactual outcome *y*_*s*_0__. This is different than the normal counterfactual fairness calculation in Definition 3.3, which requires the prediction to be equal across the factual/counterfactual pairs, without caring if those pairs have the same outcome prediction. Therefore, in addition to requiring predictions to be equal across factual/counterfactual samples, those samples must also share the same value of the actual outcome *Y*. In other words, it considers the desiderata from both counterfactual fairness and equalized odds. For our running example, this is an extension of the discussion under Definition 3.3 in which we now require that ŷ_*s*_0__ = ŷ_*s*_1__.

### 3.11. Fair on Average Causal Effect

In Khademi et al. (2019), Khademi et al. introduced two definitions of group fairness: fair on average causal effect (FACE), and fair on average causal effect on the treated (FACT) based on the Rubin-Neyman potential outcomes framework. Let *Y*_*i*_(*s*) be the potential outcome of an individual data point *i* had *S* been *s*.

**Definition 3.17 [Fair on Average Causal Effect (FACE)]**. *A decision function is said to be fair, on average over all individuals in the population, with respect to* *S*, *if* 𝔼[*Y*_*i*_(*s*_1_) − *Y*_*i*_(*s*_0_)] = 0.

FACE considers the average causal effect of the marginalization attribute *S* on the outcome *Y* at the population level and is equivalent to the expected value of the TCE(*s*_1_, *s*_0_) in the structural causal model.

**Definition 3.18 [Fair on Average Causal Effect on the Treated (FACT)]**. *A decision function is said to be fair with respect to* *S*, *on average over individuals with the same value of* *s*_1_, *if* 𝔼[*Y*_*i*_(*s*_1_) − *Y*_*i*_(*s*_0_) ∣ *S*_*i*_ = *s*_1_] = 0.

FACT focuses on the same effect at the group level. This is equivalent to the expected value of *ETT*_*s*_1_, *s*_0__(*Y*). The authors used inverse probability weighting to estimate FACE and use matching methods to estimate FACT.

### 3.12. Equality of Effort

In Huang et al. (2020), Huang et al. developed a fairness notation called equality of effort. When applied to the example in Figure 4, we have a dataset with *N* individuals with attributes (*S, T*, **X**, *Y*) where *S* denotes the marginalization attribute gender with domain values {*s*^+^, *s*^−^}, *Y* denotes a decision attribute admission with domain values {*y*^+^, *y*^−^}, *T* denotes a legitimate attribute such as test score, and **X** denotes a set of covariates. For an individual *i* in the dataset with profile (*s*_*i*_, *t*_*i*_, **x**_*i*_, *y*_*i*_), they may ask the counterfactual question, how much they should improve their test score such that the probability of their admission is above a threshold γ (e.g., 80%).

**Definition 3.19 [γ-Minimum Effort]**. *For individual* *i* *with value* (*s*_*i*_, *t*_*i*_, **x**_*i*_, *y*_*i*_), *the minimum value of the treatment variable to achieve γ-level outcome is defined as*:


(36) $$\begin{array}{l} {\text{Ψ}}_{i}\left(\gamma \right)=\underset{t\in T}{\mathrm{argmin}}\{𝔼[Y_{i}(t)]\geq \gamma )\} \end{array}$$

*and the minimum effort to achieve γ-level outcome is* Ψ_*i*_(γ) − *t*_*i*_.

If the minimal change for individual *i* has no difference from that of counterparts (individuals with similar profiles except the marginalization attribute), individual *i* achieves fairness in terms of equality of effort. As *Y*_*i*_(*t*) cannot be directly observed, we can find a subset of users, denoted as *I*, each of whom has the same (or similar) characteristics (**x** and *t*) as individual *i*. I^*^ denotes the subgroup of users in *I* with the marginalization attribute value s^*^ where * ∈ {+, −} and $𝔼\left[Y_{I^{*}}\left(t\right)\right]$ denotes the expected outcome under treatment *t* for the subgroup I^*^.

**Definition 3.20 [γ-Equal Effort Fairness]**. *For a certain outcome level γ, the equality of effort for individual* *i* *is defined as*:


(37) $$\begin{array}{l} {\text{Ψ}}_{I^{+}}\left(\gamma \right)={\text{Ψ}}_{I^{-}}\left(\gamma \right). \end{array}$$

*where* ${\text{Ψ}}_{I^{*}}\left(\gamma \right)={\mathrm{argmin}}_{t\in T}\left\{𝔼\left[Y_{I^{*}}\left(t\right)\right]\geq \gamma \right\}$ *is the minimal effort needed to achieve γ level of outcome variable within the subgroup* * ∈ {+, −}.

Equal effort fairness can be straightforwardly extended to the system (group) level by replacing *I* with the whole dataset *D* (or a particular group). Different from previous fairness notations that mainly focus on the effect of the marginalization attribute *S* on the decision attribute *Y*, the equality of effort instead focuses on to what extend the treatment variable *T* should change to make the individual achieve a certain outcome level. This notation addresses the concerns whether the efforts that would need to make to achieve the same outcome level for individuals from the marginalized group and the efforts from the non-marginalized group are different. For instance, if we have two students with the same credentials minus their gender, and the Female student was required to raise their test score significantly more than the Male, then we do not achieve equal effort fairness.

### 3.13. Technical Pitfalls of Causality-Based Fairness

Causality provides a conceptual and technical framework for measuring and mitigating unfairness by using the causal effect on a decision from hypothetical interventions on marginalization attributes such as gender. Despite the benefits of causality-based notions over statistical-based ones, there have been technical challenges in applying causality for fair machine learning in practice. One common challenge is the validity of the assumptions in causal modeling. As discussed in Section 3, the majority of research on causal fairness is based on SCM which represents the causal relationships between variables *via* structural equations and a directed acyclic graph (DAG). In practice, learning structural equations and constructing the DAG model from observational data is a challenging task and often relies on strong assumptions such as the Markov property, faithfulness, and sufficiency (Glymour et al., 2019). Simply speaking, the Markov property requires that all nodes are independent of their non-descendants when conditioned on their parents; faithfulness requires all conditional independent relationships in the true underlying distribution are represented in the DAG; and sufficiency requires any pair of nodes in the DAG has one common external cause (confounder). These assumptions help narrow down the model space, however, they may not hold in the causal process or the sampling process that generates the observed data.

Another common challenge of causality-based fairness notions based on SCMs is identifiability, i.e., whether they can be uniquely measured from observational data. As causality-based fairness notions are defined based on different types of causal effects, such as total effect on interventions, direct/indirect discrimination on path-specific effects, and counterfactual fairness on counterfactual effects, their identifiability depends on the identifiability of these causal effects. Unfortunately, in many situations these causal effects are unidentifiable. Hence identifiability is a critical barrier for causality-based fairness to be applied to real applications. In the causal inference field, researchers have studied the reasons for unidentifiability and identified the corresponding structural patterns such as the existence of the “kite graph”, the “w graph”, or the “hedge graph”. We refer readers who are interested in learning the specifics of identifiability theory and criteria, and how they can be used to decide the applicability of causality-based fairness metrics to Makhlouf et al. (2022). We also refer readers to Wu et al. (2019) for a summary of unidentifiable situations and approximation techniques to derive bounds of causal effects.

The potential outcome framework does not require the causal graph. However, as discussed in Section 2.3, it relies on three assumptions. SUTVA is a non-interference assumption which may not hold in many real world applications. For example, a loan officer's decision to proceed with one application may be influenced by previous applications. In this case, SUTVA is violated. When the strong ignorability assumption does not hold, there exist hidden confounders. Although we can leverage mediating features or proxies to estimate treatment effects (Miao et al., 2018), the lack of accuracy guarantee hinders the applicability of causal fairness.

## 4. Philosophy of Causality

The first formal investigation into causality was done by the Greek philosopher Aristotle, who in 350 BC, published his two famous treatise, *Physics* and *Metaphysics*. In these treatise, Aristotle not only opposed the previously proposed notions of causality for not being grounded in any solid theory (Falcon, 2019), but he also constructed a taxonomy of causation which he termed “the four causes.” In order to have proper knowledge, he deemed that we must have grasped its cause, and that giving a relevant cause is necessary and sufficient in offering a scientific explanation. His four causes can be seen as the four types of answers possible when asked a question of “why.”

1. The material cause: “that out of which” (something is made). E.g., the marble of a statue.
2. The formal cause: “the form”, “the account of what-it-is-to-be.” E.g., the shape of the statue.
3. The efficient cause: “the primary source of the change or rest.” E.g., the artist/sculptor of the marble statue.
4. The final cause: “the end, that for the sake of which a thing is done.” E.g., the creation of a work of art.

Despite giving four causes, Aristotle was not committed to the idea that every explanation had to have all four. Rather, he reasoned that any scientific explanation required *up to* four kinds of cause (Falcon, 2019).

Another important philosopher who worked on causality was the 18th century Scottish philosopher David Hume. Hume rejected Aristotle's taxonomy and instead insisted on a single definition of cause. This is despite the fact that he himself could not choose between two different, and later found to be incompatible, definitions (Pearl and Mackenzie, 2018). In his *Treatise of Human Nature*, Hume states that “several occasions of everyday life, as well as the observations carried out for scientific purposes, in which we speak of a condition A as a cause and a condition B as its effect, bear no justification on the facts, but are simply based on our habit of observing B after having observed A” (Frosini, 2006). In other words, Hume believed that the cause-effect relationship was a sole product of our memory and experience (Pearl and Mackenzie, 2018). Later, in 1739, Hume published *An Enquiry Concerning Human Understanding* in which he framed causation as a type of correlation: “we may define a cause to be an object followed by another, and where all the objects, similar to the first, are followed by objects similar to the second. Or in other words, where, if the first object had not been, the second never had existed.” While he tried to pass these two definitions off as one by using “in other words,” David Lewis pointed out that the second statement is contradictory to the first as it explicitly invokes the notion of a counterfactual which, cannot be observed, only imagined (Pearl and Mackenzie, 2018).

**Figure 6:**
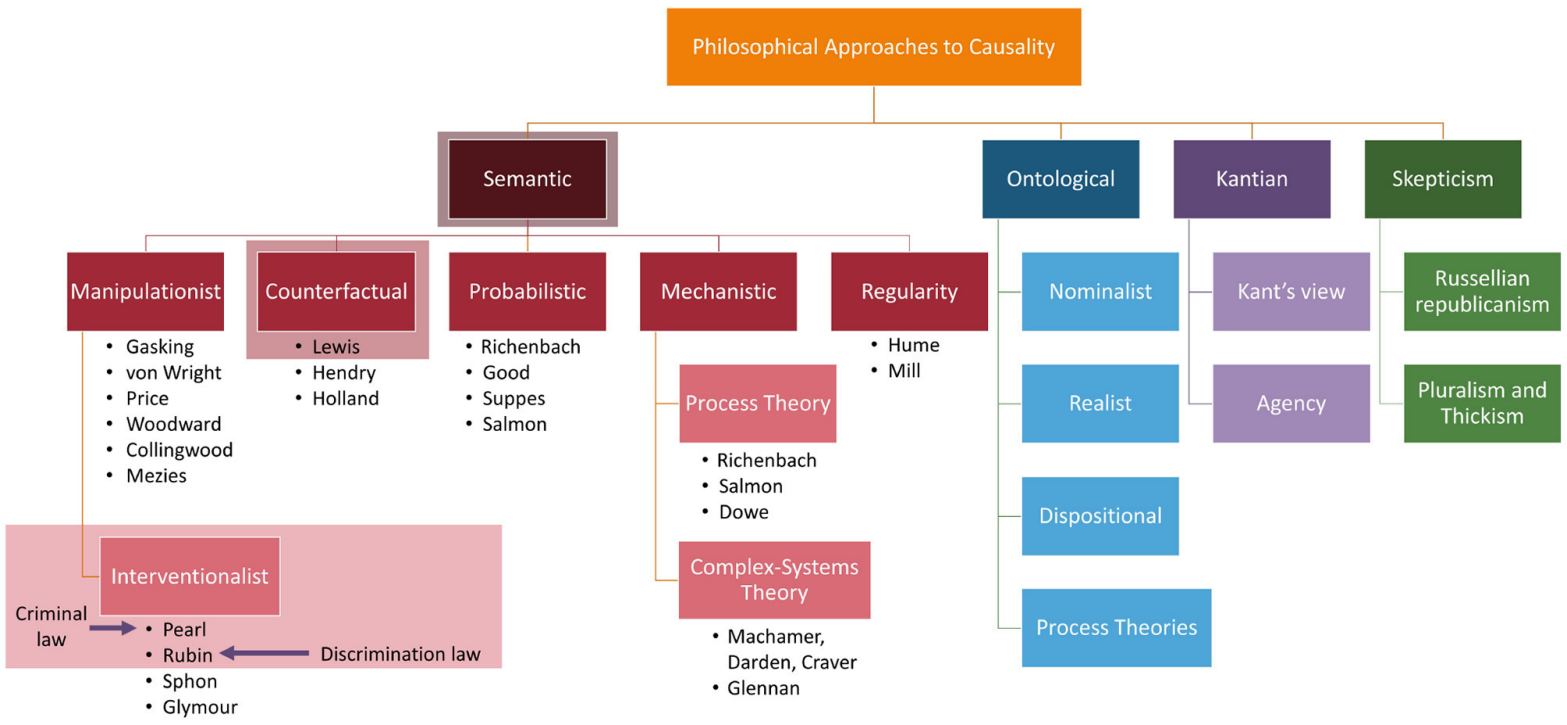
The multiple different approaches to causality. The first level indicates the types of questions that can be asked about causality and the rows below are answers to the questions. For example, probabilistic approaches are an answer to semantic questions. The shadow-boxed items are elements discussed in this publication: interventionalist approaches of Pearl and Rubin as well as the counterfactual groundings of both based on Lewis' work. Additionally, we map where two different types of U.S. legal cases fall within the causal framework. We refer interested readers to Broadbent (2020) for details on the approaches not covered.

It is also important to note that Hume changed how philosophers approached causality by changing the question from “What is causality” to “What does our concept of causality mean?” In other words, he took a metaphysical question and turned it into an epistemological one^8^ (Broadbent, 2020). This change allowed philosophers to take different approaches to answering the new question such as those based on semantic analyses, ontological stances, skepticism, and Kantian stances. Each of these approaches in turn garnered several theories of how to formulate an answer. A breakdown of all the approaches and theories can be seen in Figure 6.

Since this publication is focused on the SCM and PO frameworks, we will mainly constrain our analysis to the interventionalist theories as well as a brief discussion of the counterfactual theory by David Lewis since they are closely related. Additionally, we will give a short overview of each theory type to answering “What does our concept of causality mean” from a semantic approach to give insight into why the theories of Pearl and Rubin from an interventionalist approach are popular in causality-based machine learning fairness notions.

### 4.1. Regularity

The regularity theory implies that causes and effects do not usually happen just once, rather they happen as part of a regular sequence of events. For instance, today it rained causing the grass to be wet, but rain, no matter the day, produces this effect. This theory claims that in order to firmly say that one event causes another it must be that the cause is followed by the effect and that this cause-effect pair happens a lot. In other words, the cause and effect must be constantly conjoined (Broadbent, 2020). Hume's definition of causality from *An Enquiry Concerning Human Understanding* is a well-regarded regularity theory.

### 4.2. Mechanistic

The mechanistic theory of causality says that explanations proceed in a downward direction: given an event to be explained, its mechanism (cause) is the structure of reality that is responsible for it (Williamson, 2011). I.e., two events are causally connected if and only if they are connected by an underlying physical mechanism. This is in contrast to causal explanations which often operate in a backwards direction: given an event to be explained, its causes are the events that helped produce it. There are two main kinds of mechanistic theory: 1) process theory which says that *A* causes *B* if and only if there is a physical process (something that transmits a mark or transmits a conserved physical quantity like energy-mass) that links *A* and *B*; and complex-system theory which says that *A* and *B* are causally related if and only if they both occur in the same complex-system mechanism (a complex arrangement of events that are responsible for some final event or phenomenon because of how the events occur).

### 4.3. Probabilistic

Probabilistic theories operate under the assumption that a cause occurring raises the probability of their corresponding effects. For example, “striking a match may not always be followed by its lighting, but certainly makes it more likely; whereas coincidental antecedents, such as my scratching my nose, do not” (Broadbent, 2020). Probabilistic theories of causality are motivated by two main notions: 1) changing a cause makes a difference to its effects; and 2) this difference shows up in the probabilistic dependencies between the cause and effect (Williamson, 2009). Additionally, many probabilistic theorists go further and say that probabilistic dependencies provide necessary and sufficient conditions for causal connections. Further, many go one step farther and say that probabilistic dependencies give an analysis of a causal relation. I.e., that *C* causes *E* simply means that the corresponding probabilistic dependencies occur.

### 4.4. Counterfactual

David Lewis's counterfactual theory of causation (Lewis, 1973) starts with the observation that if a cause had not happened, the corresponding effect would not have happened either. A cause, according to Lewis in his 1973 article “Causation”, was “something that makes a difference, and the difference it makes must be a difference from what would have happened without it” (Hidalgo and Sekhon, 2011). He defined causal inference to be the process of comparing the world as it is with the closest counterfactual world. If *C* occurs both in the actual and the closest counterfactual world without *A*, then it must be that A is not the cause of *C*. Many note that since he provided sparse practical guidance on how to construct counterfactual worlds, his theories when used alone have limited use to empirical research (Hidalgo and Sekhon, 2011).

Additionally, it may seem odd that counterfactuals constitute a whole separate theory and is not combined with the manipulation or interventionalist theories. But this is because interventionalist theories shift the approach from pure conceptual analysis to something more closely related to causal reasoning and focused on investigating and understanding causation than producing a complete theory (Broadbent, 2020). This point additionally highlights why interventionalist approaches to both causal frameworks and causality-based machine learning fairness metrics are popular. The PO and SCM frameworks of Rubin and Pearl have risen to the forefront since their treatment of causality is no longer purely theoretical. They give tools and methods to actually implement causality-based notions rather than just speak to “what does our concept of causality mean.” One might say that the probabilistic theories technically gave a mathematical framework for a possible implementation, but in reality, they did not produce any new computational tools or suggest methods for finding causal relationships and so were abandoned for using interventionalist approaches instead (Hitchcock, 2021).

### 4.5. Manipulation and Interventionalist

Manipulability theories equate causality with manipulability. In these cases, *X* causes *Y* only when you can change *X* in order to change *Y*. This idea makes intuitive sense with how we think about causation since we often ask causal questions in order to change some aspect of our world. For instance, asking what causes kids to drop out of school so that we might try to increase retention rates. But, most philosophical discussion on manipulability theories have been harsh. Two complaints have been that manipulability theories are circular in nature and that they produce theories that are not valid since it depends on being able to actually manipulate the variable at hand to cause an effect, i.e., changing the race of a person to observe if the final effect differed (Woodward, 2016). The interventionist framework was proposed to overcome these issues and to present a plausible version of a manipulability theory.

Interventionalist approaches attempt to perform a surgical change in *A* which is of such a character that if any change occurs in *B*, it occurs only as a result of its causal connection to *A*. In other words, the change in *B*, that is produced by the surgical change of *A* should be produced only *via* a causal path that goes through *A* (Woodward, 2016). Both Judea Pearl and Donald Rubin have interventional theories - Rubin in the PO framework and Pearl in the SCM framework. Pearl noted that causal events can be formally represented in a graph which enabled the display of the counterfactual dependencies between the variables (Pearl, 2009; Pearl and Mackenzie, 2018). The counterfactual dependencies are then analyzed against what would happen if there was an (hypothetical) intervention to alter the value of only a specified variable (or variables). Pearl suggested that formulating causal hypotheses in this manner offered the mathematical tools for analyzing empirical data (Broadbent, 2020).

In contrast with Pearl, Rubin advocated for the treatment of causation in terms of more manipulation-based ideas, meaning that causal claims involving causes that are un-manipulable in the principle are defective (Woodward, 2016). Un-manipulable does not mean variables that cannot be manipulated due to practical reasons, but rather variables that do not have a clear conception of what it would take to manipulate them, such as race, species, and gender.

## 5. Causality and the Law

As we briefly showed in Figure 6, both U.S. discrimination law and criminal law can be mapped to frameworks that belong to the interventionalist theories of Pearl and Rubin. Below we will discuss each of these types of case law in relation to causality-based fair machine learning in more detail in order to show how the research on causal framework, and more importantly, causality-based fair machine learning, is put to work in practical scenarios.

### 5.1. Discrimination Law

In discrimination law, there are two main types of cases: 1) disparate impact (DI) cases in which there is unintentional or indirect discrimination; and 2) disparate treatment (DT) in which an individual is intentionally treated different based on their membership in a marginalized class. In this publication we will center our focus on DT since DI is often associated with statistical parity. Causation, in the legal sense of the word, is the element of a legal claim that connects a defendant's actions to a plaintiff's (i.e., victim's) injury or wrongdoing. In both types of cases, the most prevalent ‘standard' of causation is the “but-for” standard.

The but-for test says that a defendant's action is a but-for cause of the plaintiff's harm if, were it the case that the defendant didn't carry out the action, then the harm would not have occurred. While the but-for test is a straightforward test that aligns with our notion of common sense, it has been found that in many disparate treatment cases the but-for case is an inadequate measure of causation. This is because the majority of discrimination cases are a *mixed-motive* claims—claims in which there are at least two possible motives that lead to the action where one motive is discriminatory and one is not (Bavli, 2021). The mixed-motive claims make it difficult to find the defendant's true motive, and, therefore, the defendant can easily win the case by presenting evidence of a legitimate purpose for the action (Bavli, 2021).

Resulting from issues with the but-for test in DT cases, the “motivating-factor” test was created. The motivating-factor test has one simple requirement: the ruling will be in favor of the plaintiff if and only if they can show that a discriminatory reason was a *motivating* factor in the decision. Unfortunately, this test strays away from actual cause and effect, its meaning is vague even to judges and court since ‘motivating factor’ is not defined, and it allows the jury to rely on simple intuition to decide if the defendant's action was based on the presented evidence of discrimination (Bavli, 2021).

Since historically there has been an inconsistent use of the two tests, and each test has steep downfalls, many call for a total overhaul of the notion of causality in the legal field. More specifically, many propose to use the PO framework to determine if the defendant's actions caused the plaintiff's harm (Foster, 2004; Greiner, 2008; Bavli, 2021). This is because the PO framework allows for an understanding and application of causation that is broader than the ‘causation’ of the but-for tests, but it still retains the use of a necessity condition (see Pearl, 2009 for more information on necessary and sufficient causes) (Bavli, 2021). Not only would utilizing the PO framework clear the confusion present in the two tests, but it would also allow the courts, litigators, and jury to better understand the causal problem at hand to determine if the defendant is to blame or not. Another reason why a focus on using the PO framework is emerging is because statistical-based fairness metrics do not align well with DT cases since discrimination claims usually require plaintiffs to demonstrate a causal connection between the challenged decision and the marginalization attribute.

Continued research in causality-based fair machine learning notions will only strengthen the support of use of PO in DT cases. This is because these notions, without having to construct a complex causal graph, focus on estimating the causal effects of treatment (marginalization) variables on the outcome in a *consistent* manner. For example FACE and FACT (see Definitions 3.17 and 3.18) measure the effect of the marginalization variable on the outcome at both population and group levels which gives a clear measure if discrimination exists in a certain setting or not.

### 5.2. Criminal Law

While DT cases connected with the PO framework, criminal law cases can be mapped to the SCM framework. When proving guilt in a criminal court case, the prosecution is required to prove that the defendant's action was the legal cause of the result. Establishing this causal relationship is a two-step process in which the prosecution first establishes *factual* (“but-for”) causation and then determines if there is *proximate* causation (Kaplan et al., 2012).

To prove factual causation, the prosecutor does not have to prove that the defendant's actions were the sole cause of the result (such as in DT), as their actions may have been combined with those of another person, or another circumstance, that all contributed to the final result. An exception to factual causation is when the chain of events caused by the defendant's actions is effectively broken. These intervening factors must be unforseeable. For instance, if the defendant's actions put the victim in the hospital (in a non-critical condition), but by the effect of gross medical malpractice, they die, then, the defendant would most likely be charged for assault, but not homicide.

After proving factual causation, the prosecution must then prove proximate causation, which is a cause that is legally sufficient to result in liability. Typically, proximate cause issues arise when the final result occurs through an unexpected manner. For instance, if the defendant shot the victim in the arm, who then while running away from the defendant, fell on the sidewalk and cracked their skull which resulted in their death a few moments later, then the defendant's actions were the proximate cause of the victims death. The general rule is that the defendant's actions will be regarded as the proximate cause of a result if the result occurred as a “natural and probable consequence” of the acts, and there was no intervening factor sufficient enough to break the chain of causation (People v. Geiger, 1968; LawShelf, 2021).

**Figure 7:**
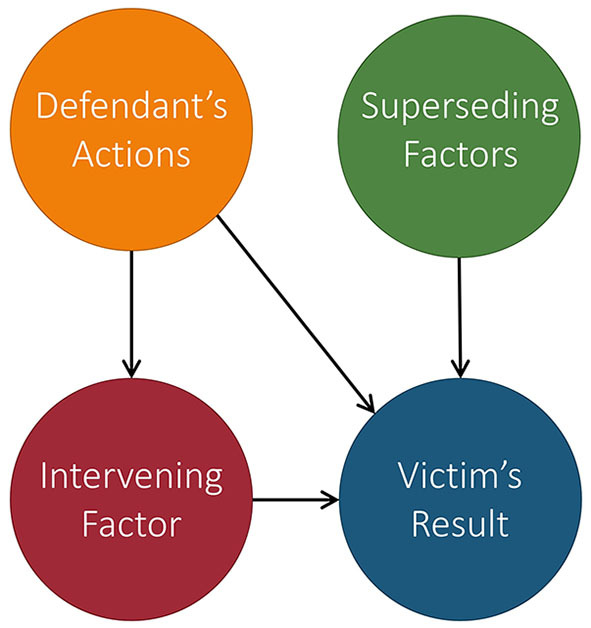
Graphical model displaying the causal relationships between the defendant's actions, the victim's result, along with intervening and superceding factors.

Using the SCM framework, we can display the relationship of causation in the law as shown in Figure 7. When relating to casual-based fairness metrics, the legal notion of causality closely aligns with the idea of path-specific causal effect. In this case, instead of computing the direct and indirect effects, path-specific causal effect isolates the contribution of the effect along a specific group of paths (Chiappa, 2019). This is similar to (but not actually) how lawyers and judges make decisions on if a certain action caused a certain effect. For instance, they reason if the intervening factor (if there is one) played a role in the victim's result and if this intervening factor “broke the chain” of the defendants actions in a way that no longer holds them liable. This would result in turning *Defendant's Actions* → *Intervening Factor* → *Victim's Result* to simply be *Intervening Factor* → *Victim's Result* as shown in Figure 7.

We note that, despite our example in the above paragraph, there is currently no formal use of SCMs (or PO) in the legal field. Additionally, while several rulings from various judges seem to invoke counterfactual language (Carson vs Bethlehem Steel Corp., 1996; Univ. of Tex. Sw. Med. Ctr. v. Nassar, 2013), there is no directive or standard that ties causality in the legal realm to causality in the causality-based machine learning realm (Barocas et al., 2019). But we hope that our discussion above spurs further conversations about combining work in causality-based fair machine learning with research in the legal field. Additionally, we hope it gives perspective on how the notions we produce could be used into practical situations.

## 6. Sociological Criticism of Causality-Based Fairness Notions

One point about causality that we have not mentioned previously is that it has a property, called *modularity*, that allows it to make causal connections. Modularity is what allows us to change the connection between any two variables while leaving the other causal relationships untouched (often in the form of the *do*-operator). For instance, changing the gender of an applicant while keeping the major the same. Modularity is a cornerstone of causal inference, but many believe it is also its downfall (Kohler-Hausmann, 2017).

To see why this is the case, we will first explain another issue critics of causal inference raise—that in order to talk about the causal effect of social categories, and to be able to manipulate them, we first need to concretely define what a social category *is* (Kasirzadeh and Smart, 2021). Many in the social science field, while not agreeing on one set definition of “group”, believe that social categories and groups extend beyond being purely genetic and rather are social constructs that depend on the experiences lived by those in it. For example, applying to a humanities department partly defines what it means to belong to the social group of Female, as does birthing a child or being more prone to injuries in a car accident (Bakalar, 2022). These critics believe that the role social categories play in structuring life experiences makes it illogical to say two individuals are exactly the same, save for their gender or race (Kohler-Hausmann, 2017), and that causality-based fairness approaches suffer from the fundamental error of thinking membership in a group is separable from the social experiences lived by those in it. In other words, the modularity property is unusable, which effectively breaks all of causal inference theory.

One solution to the above problems with causal models has been proposed by Hu and Kohler-Hausmann—an approach they termed *constitutive models* (Hu and Kohler-Hausmann, 2020). They suggest that formal diagrams of constitutive relations would allow a new line of reasoning about discrimination as they offer a model of how the meaning of a social group is formed from its constitutive features. Constitutive relations show how societal practices, beliefs, regularities, and relations make up a category (Hu and Kohler-Hausmann, 2020). They also note that causal diagrams can simply be reformatted to be constitutive ones, and that because a constitutive model provides a model of what makes a category, it presents entirely the information needed to debate about what practices are discriminatory (Hu and Kohler-Hausmann, 2020).

## 7. Conclusion

We have attempted to remedy a long standing problem in the fair machine learning field, namely, the abstraction of the technical aspects of fairness notions from their philosophical, sociological, and legal connections. By explaining the details of popular causality-based fair machine learning notions in both formal and social science terminology, ultimately, we recenter the fair machine learning discussion as one of a sociotechnical nature, rather than simply a technical one. We hope that this field guide not only helps fair machine learning practitioners understand how specific causality-based fairness notions align with long-held humanistic values, but also that it will spark conversation and collaboration with the social science field to construct better fairness notions.

## Author Contributions

AC and XW contributed to conception and design of the work. AC detailed the background, philosophy, social, and law sections. XW detailed the causal framework and metrics. All authors contributed to manuscript writing and revision, read, and approved the submitted version.

## Funding

This work was supported in part by NSF 1910284, 1920920, and 2137335.

## Conflict of Interest

The authors declare that the research was conducted in the absence of any commercial or financial relationships that could be construed as a potential conflict of interest.

## Publisher's Note

All claims expressed in this article are solely those of the authors and do not necessarily represent those of their affiliated organizations, or those of the publisher, the editors and the reviewers. Any product that may be evaluated in this article, or claim that may be made by its manufacturer, is not guaranteed or endorsed by the publisher.

## Footnotes

^1^What we call the marginalization attribute (or marginalized class) is often called the sensitive or protected attribute/class in fair machine learning literature. See our extended version on ArXiv for the specifics of why we do so.

^2^The field of Science and Technology Studies (STS) describes systems that consist of a combination of technical and social components as “sociotechnical systems” (Selbst et al., 2019).

^3^A spurious association is a relationship where two or more variables are associated, but not causally related.

^4^To learn more about structural equation modeling (SEM), see Pearl, 2009.

^5^Here, marginalization refers to marginal distributions in probability, not a “sensitive” variable.

^6^“Data is profoundly dumb. Data can tell you that people who took a medicine recovered faster than those who did not take it, but they can't tell you why.” - Judea Pearl (Pearl and Mackenzie, 2018).

^7^A backdoor path from *X* to *Y* is any path starting at *X* with a backward edge ← into *X* such as: *X* ← *A* → *B* ← *C* → *Y*. Backdoor paths allow information to flow from *X* to *Y* in a way that is not causal.

^8^Metaphysics is the study of reality, while epistemology is the study of knowledge. Epistemology looks at how we know what the truth is and whether there are limits to this knowledge, while metaphysics seeks to understand the nature of reality and existence.

## Abbreviations

ATE: Average Treatment Effect
ATT: Average Treatment effect on the Treated
CATE: Conditional Average Treatment Effect
CDE: Controlled Direct Effect
CE: Counterfactual Effect
Ctf-DE/IE/SE: Counterfactual Direct/Indirect/Spurious Effect
DI: Disparate Impact
DT: Disparate Treatment
ER/EO: Error Rate Balance
ER^*d*/*i*/*s*^: Counterfactual Direct/Indirect/Spurious Error Rate
ETT: Effect of Treatment on the Treated
FACE: Fair on Average Causal Effect
FACT: Fair on Average Causal Effect on the Treated
ITE: Individual Treatment Effect
NDE: Natural Direct Effect
NIE: Natural Indirect Effect
PCE: Path-specific Counterfactual Effect
PE: Path-specific Effect
SCM: Structural Causal Models
SUTVA: Stable Unit Treatment Value Assumption
TCE: Total Causal Effect
TV: Total Variation.
